# CXCL12 and CD3E as Indicators for Tumor Microenvironment Modulation in Bladder Cancer and Their Correlations With Immune Infiltration and Molecular Subtypes

**DOI:** 10.3389/fonc.2021.636870

**Published:** 2021-03-04

**Authors:** Yi Liu, YuCai Wu, PeiPei Zhang, ChaoJie Xu, ZeSen Liu, ChaoJie He, YiMing Liu, ZhengJun Kang

**Affiliations:** ^1^ Department of Urology, The Fifth Affiliated Hospital of Zhengzhou University, Zhengzhou University, Zhengzhou, China; ^2^ Department of Urology, Peking University First Hospital, Beijing, China; ^3^ Department of Pediatrics, The Fifth Affiliated Hospital of Zhengzhou University, Zhengzhou University, Zhengzhou, China; ^4^ Department of General Surgery, The Fifth Affiliated Hospital of Zhengzhou University, Zhengzhou University, Zhengzhou, China

**Keywords:** CXCL12, CD3E, bladder cancer, tumor microenvironment, tumor-infiltrating immune cells, prognosis, molecular subtype

## Abstract

Bladder cancer (BLCA) represents the ninth most common malignant tumor in the world and is characterized by high recurrence risk. Tumor microenvironment (TME) plays an important role in regulating the progression of BLCA. Immunotherapy, including Bacillus Calmette-Guerin (BCG) and programmed cell death protein 1 (PD-1)/programmed death ligand 1 (PD-L1), is closely associated with TME and is widely used for treating BLCA. But parts of BLCA patients have no response to these treatment ways, thus a better understanding of the complex TME of BLCA is still needed. We downloaded the gene expression profile and corresponding clinical information of 414 BLCA patients from the TCGA database. *Via* the ESTIMATE and CIBERSORT algorithm, we identified the two hub genes (CXCL12 and CD3E) and explored their correlations with immune infiltration. We found that BLCA patients with higher expression of CXCL12 and lower expression of CD3E had prolonged survival. Gene set enrichment analysis (GSEA) revealed that both CXCL12 and CD3E were enriched in immune-related pathways. We also discovered that stromal score and the level of CXCL12 were higher in luminal subtype, and immune score and the level of CD3E were higher in the basal subtype. Furtherly, we found that CXCL12 was associated with naive B cells, resting mast cell, M2 macrophages, follicular helper T cells, and dendritic cells. CD8^+^ T cells, CD4^+^ T cells, regulatory T cells (Tregs), and macrophages were correlated with CD3E. In conclusions, we found that CXCL12 and CD3E might serve as indicators of TME modulation in BLCA. Therapy targeting CXCL12 and CD3E had the potential as novel therapeutic strategy.

## Introduction

Bladder cancer (BLCA) represents the ninth most common malignant tumor in the world and the most prevalent cancer of the urinary system (1), with an excepted 81,400 newly diagnosed cases and 17,980 estimated deaths in the United States in 2020 (2). BLCA is a kind of heterogeneous cancer with various clinical outcomes. Roughly 80% of BLCA patients are non-muscle-invasive bladder cancer (NMIBC) at diagnosis (3), but about 50%–70% of the NMIBC will recur and approximately 10%–20% will develop into muscle-invasive bladder cancer (MIBC) (4). Though the improved systematic treatment, outcomes of MIBC still remain unsatisfactory (3, 5). Therefore, there is an urgent to explore novel prognostic indicators and therapeutic targets for BLCA.

Tumor microenvironment (TME) is the cellular environment in which tumor cells exist and has been proved associated with oncogenes and tumor suppression. TME is mainly composed of tumor cells and many other components, such as vascular network, the extracellular matrix (ECM), tumor-infiltrating immune cells (TICs), and also molecules for cross-talking (6, 7). Each of the ingredients has its ways to regulate the progression and metastasis of malignancies. For example, tumor cells themselves can transform the normal stem cell niches to accelerate the process of proliferation and aggressiveness (8, 9). TICs in the context of TME are closely associated with clinical outcomes. Assortment of TICs and functional interactions within TICs and tumor cells can regulate tumor development (10). For instance, M2 macrophages exhibited pro-tumoral effects through interacting with including T helper 2 cell, cancer-associated fibroblasts, cancer cells, regulatory T cells (Tregs), and myeloid-derived suppressor cells (11). In contrast, the classically activated M1 macrophages are antitumorigenic (12). A wide variety of constituent and their interlacing interaction makes the complexity of TME. Mechanisms of TME in regulating tumor progression remain unclear, but increasing evidence supports that disruption of TME may hold the potentiality of novel detection and therapeutic target.

Former researches have shown that immune and stromal signature are associated with the survival of BLCA (13, 14). Genes that may lead to TME modulation have not been well studied in BLCA and this promoted us to identify the genes inducing TME alternations.

## Material and Methods

### Gene Expression Datasets

Gene expression profiles of 433 samples, including 414 tumor tissue and 19 normal tissue, were downloaded from The Cancer Genome Atlas (TCGA) database (https://portal.gdc.cancer.gov/). The majority of BLCA patients in the TCGA database belong to MIBC. Few have variant histology or received neoadjuvant chemotherapy before. The corresponding clinical information of these patients, including age, gender, clinical stage, and pathological TNM stage, were also extracted.

### Analysis of Differentially Expressed Genes

ESTIMATE algorithm has been proved to perform well in inferring the ratio of immune and stromal components in tumor tissue (15). Stromal score, immune score, and estimate score of 414 BLCA patients were calculated through ESTIMATE algorithm. X-tile software is a tool for globally visualizing the best cut-points to parse samples into subsets (16). We used X-tile to explore the optimal cut-off value of stromal scores, immune scores, and estimate scores, and then patients were divided into high-score group and low-score group according to each cut-off value. *Via* comparing the high-score and low-score group, 381 differently expressed genes (DGEs) were identified with filter of false discovery rate (FDR) < 0.05 and |log Fold change (FC)| > 2. Heatmap of top 50 DGEs was performed by “Heatmap” R package.

### mRNA Expression Cluster Analysis

We removed genes with low abundance (expression lower than 1 in more than 25% samples) and then the top 3,000 variable genes by variance of gene expression were selected. These genes were then normalized *via* log_2_(expression+1). R package “ConsensusClusterPlus” was employed to cluster the selected genes. Basal marker genes (KRT16, KRT6C, CDH3, KRT6B, KRT14, KRT6A, CD44, KRT5, KRT1) and luminal marker genes (KRT18, ERBB2, KRT7, FOXA1, GATA3, XBP1, FGFR3, UPK1A, KRT19, KRT8, KRT20, PPARG, ERBB3, CYP2J2, UPK2, GPX2) originated from previous report (17) were used to identify the clusters and these marker genes were shown in the heatmap.

### Construction of PPI Network

The protein-protein interaction (PPI) network was constructed by String website (https://string-db.org/). Then genes with confidence of interaction score more than 0.95 were visualized with Cytoscape software (version 3.8.0).

### GO and KEGG Enrichment Analysis

To explore the potential mechanisms of DEGs, the “ClusterProfiler” R package was exploited to the Kyoto construct the Encyclopedia of Genes and Genomes (KEGG) pathway analysis and the gene ontology (GO) function analysis. Both p- and p-adjust value < 0.05 were considered significantly.

### Gene Set Enrichment Analysis

We set “c2.cp.kegg.v7.2.symbols.gmt” as our gene set database. GSEA software (version 4.1.0) was used for analysis and both NOM p value < 0.05 and FDR q value < 0.05 were considered significant.

### Tumor-Infiltrating Immune Cells Profile

The CIBERSORT method can quantify cell components from gene expression profiles of complex tissues and has been validated in former studies (18). We applied CIBERSORT to identify tumor-infiltrating immune cells (TICs) from our BLCA samples and p value < 0.05 was deemed to be statistically significant.

### Statistical Analysis

Differences between groups were compared by Wilcoxon rank sum or Kruskal–Wallis rank sum test. We performed univariate Cox regression analysis to select the genes associated with OS of BLCA patients. Hub genes were generated by the result of PPI network and univariate Cox regression analysis. The Kaplan-Meier (K-M) survival curve was drawn to demonstrate the relationship of immune score, stromal score, estimated score, hub genes, and molecular subtype with OS. The log-rank test was constructed to test the significance of the difference between groups. All statistical tests were two-sided, and P < 0.05 was considered statistically significant. All analyses were performed in R version 3.6.1 (http://www.r-project.org/) with the following packages: “limma”, “ConsensusClusterPlus”, “estimate”, “survival”, and “ClusterProfiler”.

## Results

### Immune and Stromal Components in Bladder Cancer and Their Correlations With Clinicopathologic Characteristics

**Figure 1:**
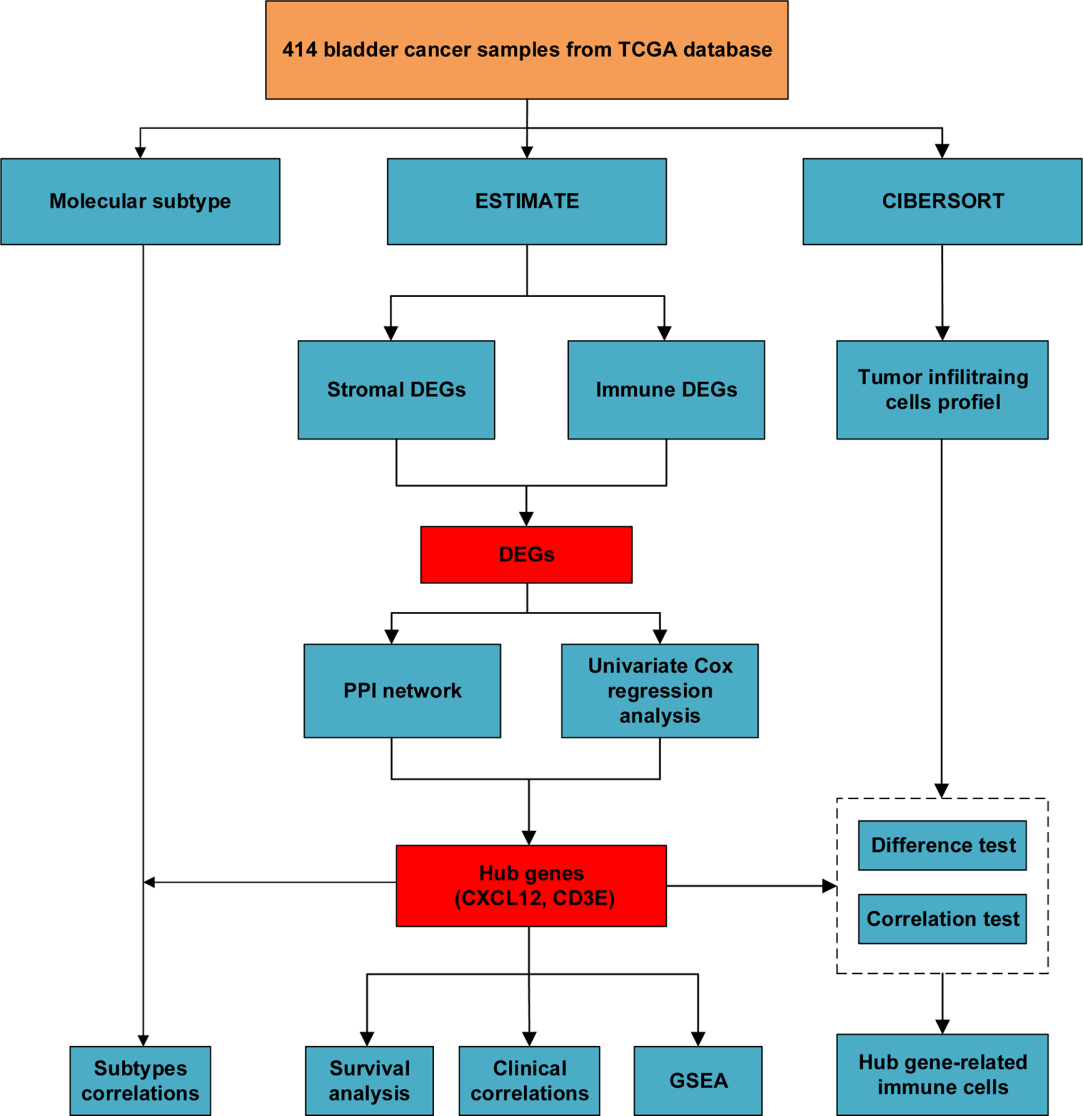
The flow chart of our study of identifying hub genes and their correlations with immune infiltration.

**Figure 2:**
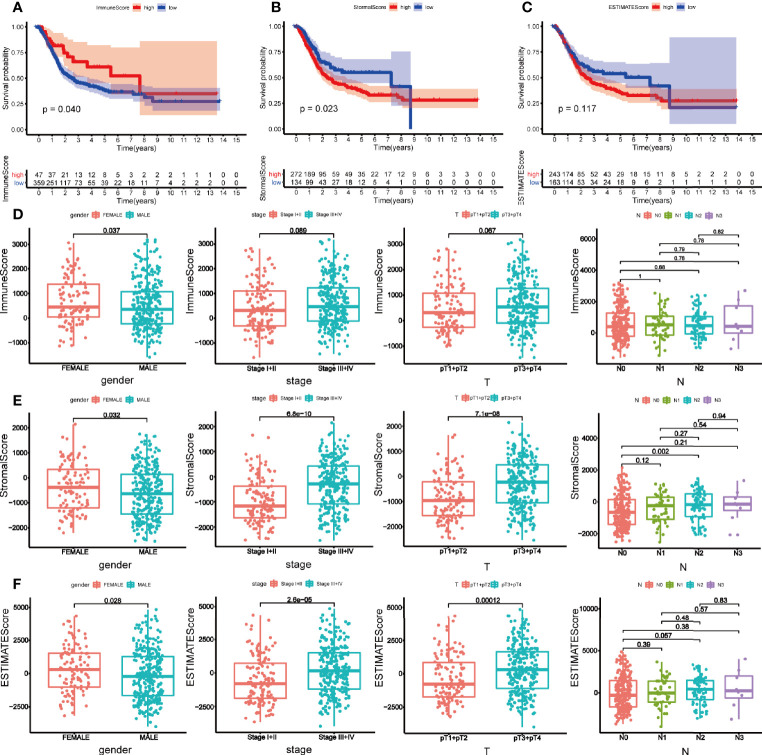
Survival analysis and clinical correlation analysis of immune, stromal, and estimated score. Kaplan-Meier analysis for overall survival of bladder cancer patients based on immune score **(A)**, stromal score **(B)**, and estimated score **(C)**. **(D)** The correlations between immune score and gender, clinical stage, pT, and N stage. **(E)** The correlations between stromal score and gender, clinical stage, pT, and N stage. **(F)** The correlations between estimated score and gender, clinical stage, pT, and N stage. P-value < 0.05 was deemed to be significant.

We downloaded 433 gene transcriptome profiles, including 414 BLCA patients and 19 normal samples from the TCGA database. The flow chart of our study was displayed in **Figure 1**. Then we calculated the immune score, stromal score, and estimate score for each patient *via* ESTIMATE algorithm. In order to explore whether immune and stromal components have impacts on the survival of BLCA patients, we divided 414 BLCA patients into two groups (high score vs low score) according to their immune, stromal, and estimate score, respectively. The cut-off points were assessed by X-tile software (**Supplementary Figure 1**). K-M analysis revealed that patients with high immune score had favorable prognosis than those with low score (**Figure 2A**). On the contrary, patients with high stromal score had shorter survival than patients with low score (**Figure 2B**). However, estimate score showed no significant correlations with OS of BLCA patients (**Figure 2C**).

Then the correlations between clinical characteristics and the components were explored. As is shown in **Figure 2**, female patients tended to get a high immune score (p = 0.037) (**Figure 2D**). Stromal score was higher in female (p = 0.032), and patients with higher clinical stage (p < 0.001) and pathological T (pT) (p < 0.001) stage obtained higher stromal score. But only difference was observed between N0 and N2 stage within N stage (p = 0.002) (**Figure 2E**). Parallel to immune and stromal score, female BLCA patients (p = 0.028), patients with higher clinical stage (p < 0.001) and pT stage (p < 0.001) got higher estimated score (**Figure 2F**).

### Differently Expressed Genes Shared by Immune and Stromal Components

**Figure 3:**
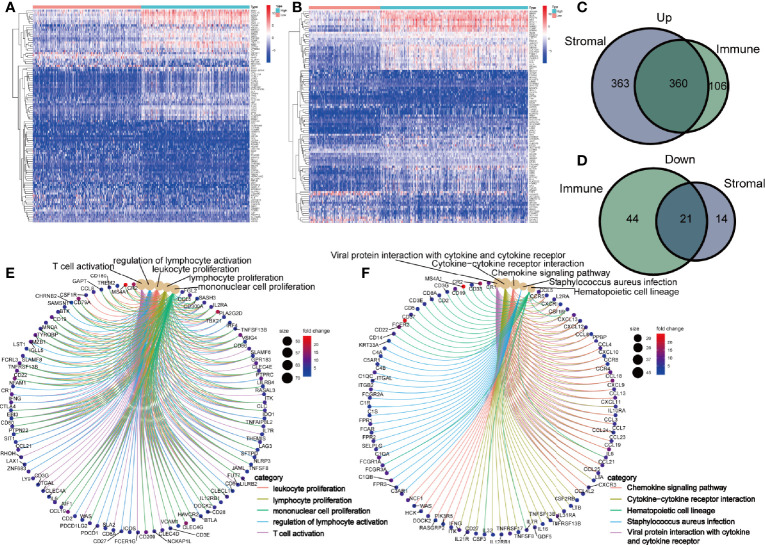
Identification of differently expressed genes (DEGs) shared by immune and stromal score. Heatmap of the top 50 DEGs in immune **(A)** and stromal components **(B)**. Venn diagram showing the shared upregulated genes **(C)** and downregulated genes **(D)** by immune and stromal components. GO **(E)** and KEGG enrichment analysis **(F)** for 381 DEGs, terms with p- and q-value < 0.05 were believed to be enriched significantly.

To identify the shared DEGs by immune and stromal components, we firstly obtained DEGs of immune and stromal components, respectively. The top 50 DEGs were displayed in heatmap (**Figures 3A, B**). By comparing the DEGs of immune and stromal components, we obtained 360 upregulated genes and 21 downregulated genes (**Figures 3C, D**). To investigate the biological classifications of these DEGs, GO enrichment analysis and KEGG pathway analysis were performed. Result of GO enrichment analysis showed that these 381 genes were mainly mapped to leukocyte proliferation, lymphocyte proliferation, mononuclear cell proliferation, regulation of lymphocyte activation, and T cell activation (**Figure 3E**). KEGG pathway analysis demonstrated that these genes were enriched in chemokine signaling pathway, cytokine-cytokine receptor interaction, hematopoietic cell lineage (**Figure 3F**).

### Identification of Hub Genes

**Figure 4:**
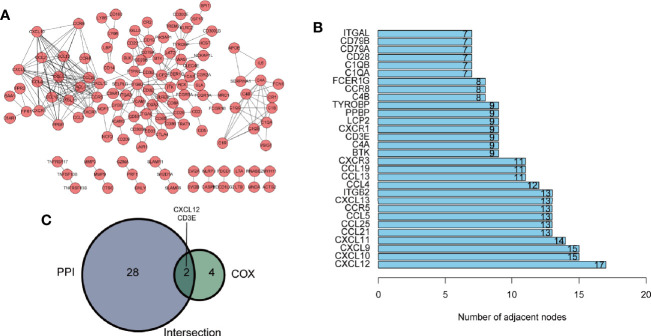
Identification of hub genes. **(A)** The protein-protein interaction (PPI) network of 381 differently expressed genes (DEGs) with interaction confidence value > 0.95. **(B)** Bar plot ranked by the edge of gene interacting. **(C)** Venn diagram of PPI network and univariate Cox regression analysis.

Hub genes were defined as prognostic genes with important role in PPI network. To pick up the hub genes from the 381 DEGs, we used the String website to construct the PPI network, which was then re-visualized in the Cytoscape software (**Figure 4A**). Nodes of gene interacting were counted and nodes of the top 30 genes were shown as bar plot (**Figure 4B**). We also performed univariate Cox survival analysis to choose the prognostic genes in BLCA and 6 genes were selected (**Table 1**). After crossing the result of the PPI bar plot and univariate Cox survival analysis, only 2 hub genes, CXCL12 and CD3E, were filtered out for further analysis (**Figure 4C**).

**Table 1:** Univariate Cox regression to identify prognostic genes of bladder cancer (BLCA).

gene	K-M	HR (95%CI)	P
TBX21	0.041	0.670 (0.497–0.905)	0.009
COMP	0.009	1.001 (1.000–1.002)	0.016
CXCL12	0.050	1.013 (1.005–1.020)	0.002
F13A1	0.019	1.005 (1.001–1.008)	0.005
MMP9	0.021	1.000 (1.000–1.000)	0.009
CD3E	0.038	0.974 (0.951–0.998)	0.032

K-M, Kaplan-Meier analysis; HR, hazard ratio; CI, confidence interval.

### Validation of Hub Genes and Their Relationship With Clinical Characteristics

**Figure 5:**
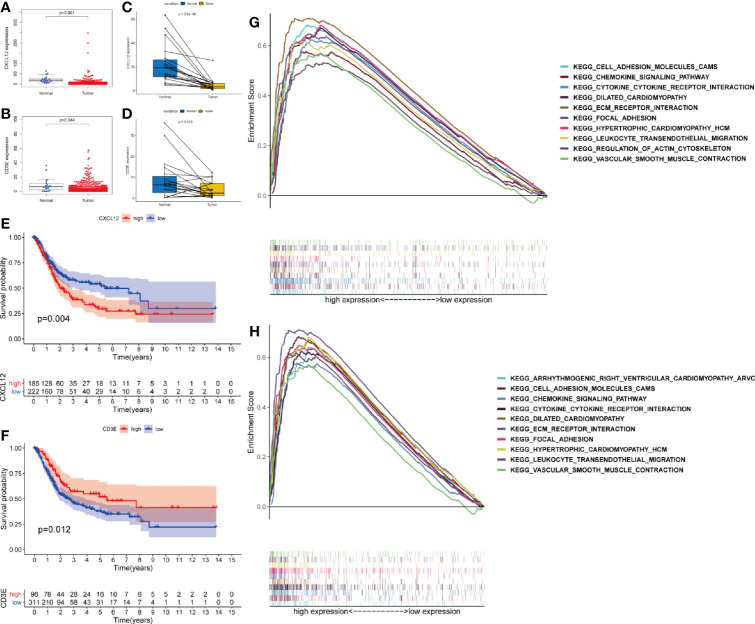
Validation the expression of CXCL12 and CD3E. Differently expressed CXCL12 **(A)** and CD3E **(B)** in normal tissue and in the tumor tissue. Similar result by paired analysis **(C, D)**. Kaplan-Meier analysis for OS of bladder cancer (BLCA) patients based on the expression of CXCL12 **(E)** and CD3E **(F)**. Gene set enrichment analysis of CXCL12 **(G)** and CD3E **(H)**.

Wilcoxon rank sum test proved that both CXCL12 (p < 0.001) and CD3E (p = 0.044) were lower in BLCA patients compared with normal patients (**Figures 5A, B**). The same results were also observed by paired analysis (**Figures 5C, D**). Once again, we used X-tile software to find the best cut-off value (**Supplementary Figure 2**). Based on the expression level of CXCL12 and CD3E, patients were divided into high-expression and low-expression group according to the cut-off value. We found that patients with higher expression of CXCL12 had worse prognosis (p = 0.004) (**Figure 5E**). However, patients with higher expression of CD3E had improved survival (p = 0.012) (**Figure 5F**). Furtherly, GSEA demonstrated that pathways in CD3E and CXCL12 high-expression group were much in common. Genes in CD3E and CXCL12 high-expression group were mainly enriched in chemokine signaling pathway, cytokine-cytokine receptor interaction, ECM receptor interaction, and leukocyte transendothelial migration (**Figures 5G, H**). These pathways were closely associated with TME regulating in BLCA. However, no gene set was enriched in CD3E or CXCL12 low-expression group.

**Figure 6:**
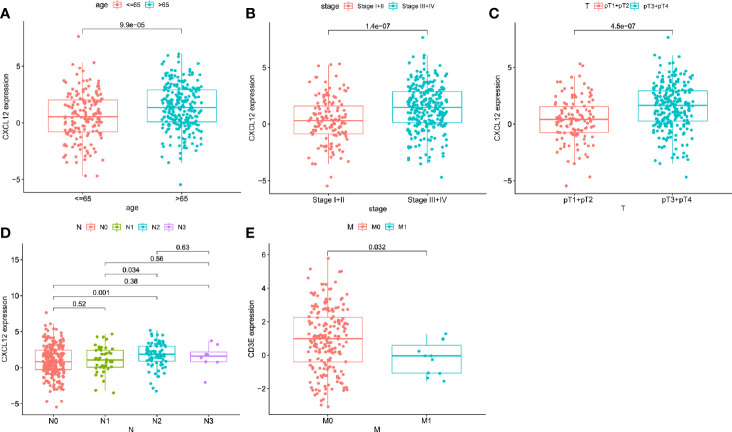
The correlations between hub genes and clinical characteristics. The correlation of CXCL12 with age **(A)**, clinical stage **(B)**, pT **(C)**, and N stage **(D)**. The correlation of CD3E with and M stage **(E)**.

The relevance of the clinical characteristics with the hub genes (CXCL12 and CD3E) was also analyzed. The level of CXCL12 was statistical difference in age (p < 0.001), clinical stage (p < 0.001), and pT stage (p < 0.001) (**Figures 6A–C**) and N2 stage owned higher level of CXCL12 than N0 (p = 0.001) and N1 stage (p = 0.034) (**Figure 6D**). Only M stage was related to the expression of CD3E (p = 0.032) (**Figure 6E**).

### Correlations of TME and Hub Genes With Molecular Subtype

**Figure 7:**
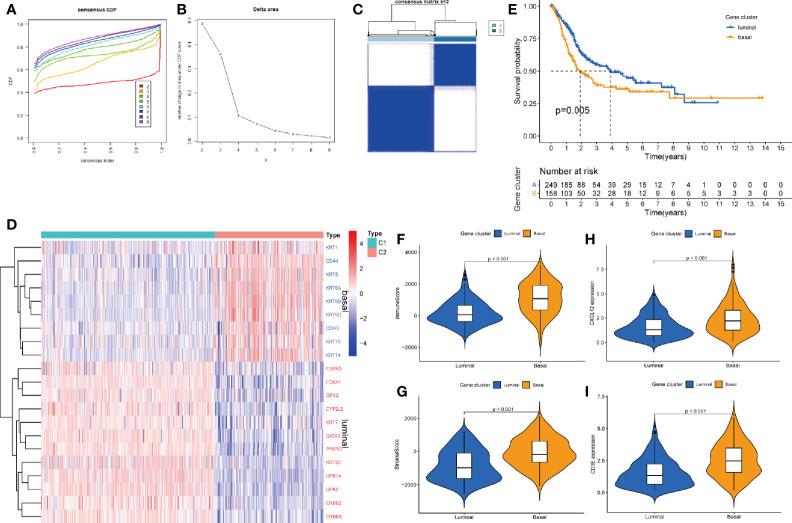
The correlations between hub genes and molecular subtypes. **(A)** Consensus clustering cumulative distribution function (CDF) for k = 2–9. **(B)** Relative change in area under CDF curve for k = 2–9. **(C)** Consensus matrix plot when k = 2. **(D)** Heatmap of luminal and basal marker genes within subtypes. **(E)** K-M analysis for OS of BLCA patients based on different subtypes. The different immune **(F)** and stromal score **(G)** in basal and luminal subtype. The different level of CXCX12 **(H)** and CD3E **(I)** in basal and luminal subtype.

After pro-processed, the top 3000 variable genes were subjected to consensus clustering analyze and the optimal cluster was obtained when k = 2 (**Figures 7A–C**). As basal and luminal subtype have been widely identified in BLCA, we analyzed the level of basal and luminal marker genes to define the two clusters. We found that basal marker genes were highly expressed in cluster 2 and luminal marker genes were highly expressed in cluster 1 (**Figure 7D**). Thus, we termed cluster 1 and 2 as luminal (n = 253) and basal (n = 161) subtype, respectively. K-M analysis showed that the luminal BLCA patients had longer survival than patients with basal subtype (**Figure 7E**). Further tests showed that the basal subtype got higher immune and stromal score than luminal subtype (**Figures 7F, G**). Both the levels of CXCL12 and CD3E were higher in the basal subtype (**Figures 7H, I**).

### Hub Genes-Related Immune Cells

**Figure 8:**
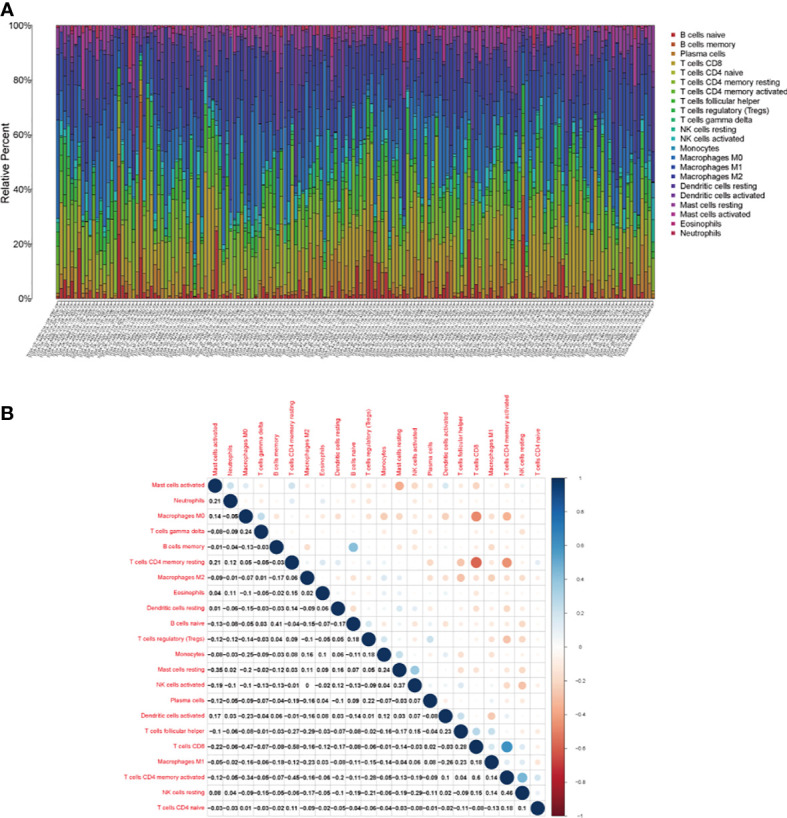
The tumor-infiltrating immune cells in bladder cancer. **(A)** The proportion of 21 kinds of tumor-infiltrating immune cells and **(B)** their correlations in bladder cancer.

To further confirm the relationship between hub genes and TME, we firstly analyzed the abundance ratio of immune cells in BLCA samples. The proportion of 22 immune cells in BLCA samples and their correlations were assessed by CIBERSORT method (**Figures 8A, B**).

**Figure 9:**
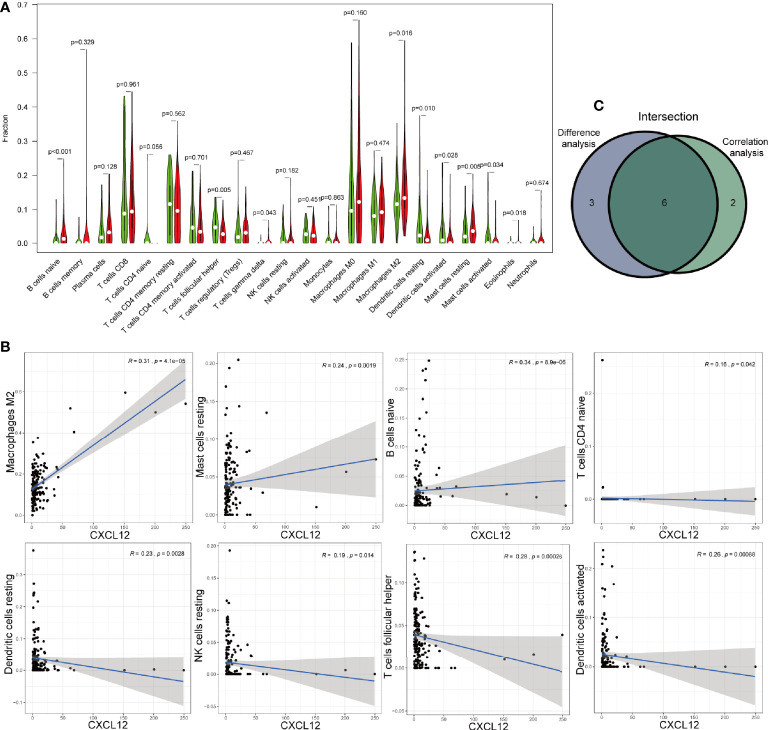
Correlations of CXCL12 with immune infiltration. **(A)** The different level of 21 kinds of tumor-infiltration cells (TICs) in CXCL12 high-expression and low-expression group. **(B)** TICs correlated with CXCL12 with p-value >0.05, including naïve B cells, resting mast cell, M2 macrophages, follicular helper T cells, resting dendritic cells, and activated dendritic cells. **(C)** Venn diagram showed the intersection of difference and correlation analysis.

Then, we analyzed the different exiting immune cells between high-expression and low-expression group. There were significant differences in nine kinds of immune cells between CXCL12 high-expression and low-expression group (**Figure 9A**). Spearman correlation test revealed that CXCL12 was associated with eight kinds of immune cells (**Figure 9B**). When merged together the different TICs with the correlated TICs, we found that naive B cells, resting mast cell, and M2 macrophages were positively associated with CXCL12, while follicular helper T cells, resting dendritic cells, and activated dendritic cells were negatively associated with CXCL12 (**Figure 9C**).

**Figure 10:**
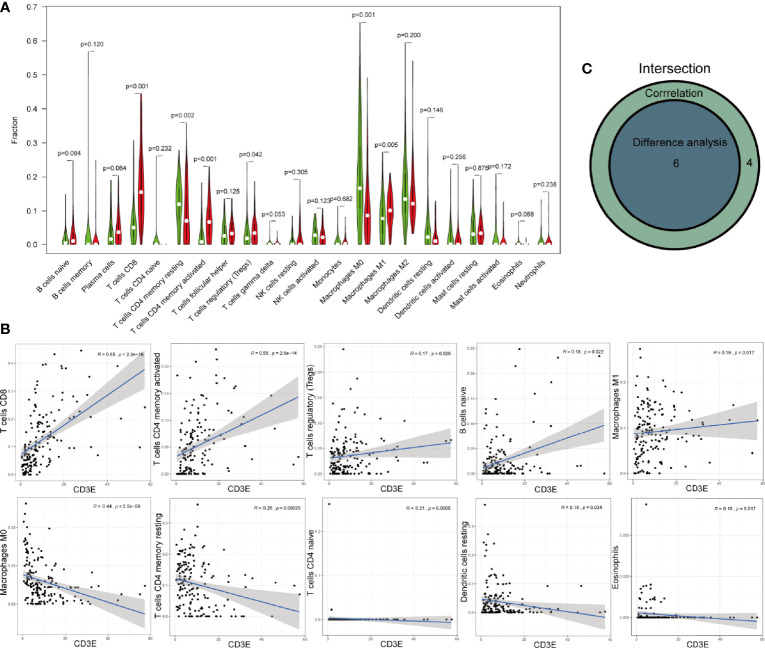
Correlations of CXCL12 with immune infiltration. **(A)** The different level of 21 kinds of tumor-infiltrating cells (TICs) in CD3E high-expression and low-expression group. **(B)** TICs correlated with CD3E with p-value >0.05, including naïve B cells, resting mast cell, M2 macrophages, follicular helper T cells, resting dendritic cells, and activated dendritic cells. **(C)** Venn diagram showed the intersection of difference and correlation analysis.

As for CD3E, we found six kinds of TICs showed significant difference between CD3E high-expression and low-expression group, which were overlapped by result of correlation analysis (**Figures 10A–C**). Four kinds of TICs, CD8^+^ T cells, CD4^+^ memory activated T cells, regulatory T cells (Tregs), and M1 macrophages, were positively associated with CD3E. Whereas, CD4^+^ memory resting T cells and M0 macrophages were negatively correlated with CD3E.

## Discussion

BLCA, as the most common malignancy of the urinary system, is characterized by its high morbidity and high incidence of recurrence. However, the mechanisms of tumorigenesis and progression of BLCA still remain unclear. TME plays an important role in many solid tumors, as well as BLCA. Intravesical therapy with Bacillus Calmette–Guerin (BCG) has been used to treat BLCA for decades but mechanism of its action is not yet fully understood. The generally accepted theory is that BCG causes a strong innate immune response involving different immune cell subsets (19, 20). Immunotherapy targeting programmed cell death protein 1 (PD-1) or its ligands, programmed death ligand 1 (PD-L1) can improve the survival of BLCA patients, but not all patients respond to this kind of therapy (21). These shreds of evidence demonstrate the importance of TME in BLCA, but to fully understand the process of BLCA, a deeper knowledge of the role of TME in BLCA is needed. Therefore, we explored the role of TME in BLCA and the genes related to TME modulation.

In this study, we downloaded 414 BLCA cases from the TCGA database. Through ESTIMATE algorithm, we found that higher immune infiltration had improved survival, whereas higher stromal infiltration was associated with shorter OS. Contrary to our results, some studies showed there was no significant impact of stromal and immune scores on the survival of BLCA patients (22, 23). The contrasted result was obtained because of the different cut-off value. We used X-title software for optimizing the best cut-off value but former researchers chose the media score as the cut-off value. Our results also supported by former studies that higher immune infiltration in MIBC is associated with improved disease-specific survival (DSS) after trimodal therapy, whereas higher stromal infiltration is associated with shorter DSS after neoadjuvant chemotherapy and radical cystectomy (14). We also found that neither immune score, stromal score or estimated score of females were higher than males. Former studies revealed that although the probability of developing BLCA in man was three to four times higher than in woman, survival of woman was worse (24). The difference existing in TME raised the possibility that TME might partly responsible for driving gender disparity in BLCA.

We identified two hub genes (CXCL12 and CD3E) which were related to TME modulation. And we found that patients with high CXCL12 showed a poor prognosis and patients with high CD3E showed a better prognosis. Similar result was also observed by a previous study (23) while we further analyzed the correlations of hub genes with TICs and molecular subtype in BLCA. CXCL12, also known as stromal cell-derived factor-1 (SDF-1), is a crucial chemokine involved in physiological and pathological processes, including embryogenesis, neurogenesis, hematopoiesis, angiogenesis, lymphopoiesis, and inflammation (25). CXCL12 can function by activating CXC chemokine receptor 4 (CXCR4), atypical chemokine receptor 3 (ACKR3), and glycosaminoglycans (26). Among receptors of CXCL12, CXCR4 can specifically bind to CXCL12 and the role of CXCL12/CXCR4 axis is most broadly explored in cancers. Interaction with this axis has emerged as being of particular interest and relevance in pancreatic cancer (27), cervical cancer (28), colorectal cancer (29), breast cancer (30), and so on. In BLCA, CXCL12/CXCR4/β−catenin pathway could contribute to proliferation, colony formation, migration, and invasion (31). Former researches also showed that CXCL12/CXCR4 axis could regulate bladder carcinogenesis *via* activating extracellular signal-regulated kinase (ERK), which was subject to TXNIP negative regulation (32). Disruption of the interaction by CXCR4 antagonist, 4F-benzoyl-TE14011 (4F-bTE), could markedly inhibit BLCA cell metastatic phenotype (33). These studies suggest that CXCL12 plays an important role in BLCA and CXCL12/CXCR4 may hold potential of providing therapeutic value to patients.

CD3 molecule can bind to T cell receptor (TCR) to assemble the CD3/TCR complex which mediates TCR signaling and T cell differentiation (34, 35). One of the subunits of CD3, CD3ϵ coding by (CD3E) gene, is associated with severe immune deficiency and is frequently used as protein target of CD3 antibody (36, 37). Charlotte et al. found that head and neck squamous cell carcinoma patients with low CD3E mRNA levels showed higher risk of recurrence (38). Similar result was also observed in squamous cervical cancer that high expression of CD3E tends to have better prognosis (39). Higher CD3 infiltration exhibited improved survival of BLCA, while the correlations of CD3E and BLCA have not been investigated. However, targeting immunotherapy for BLCA using anti-CD3×B7-H3, anti-CD3×CD155, and anti-CD3xEGFR or anti-CD3xHER2 bispecific antibody may provide novel strategies for BLCA therapy (40–42).

The luminal subtype showed longer survival than the basal subtype in our study, which has been confirmed by previous reports (43, 44). And this is corresponding with the level of stromal score and CXCL12 in luminal subtype. The worse prognosis of basal subtype presumably related to the invasive and metastatic status at presentation (43). But former research showed that basal subtype was more sensitive to immunotherapy, which might be linked to the elevated expression of immune infiltration related genes (45). For example, A research reported that basal BLCA patients showed increased PD-1 and PD-L1 expression (46). Another study demonstrated that immune biomarkers, including PD-1 (CD274), PDCD1, and CD8A, were higher in basal subtype and basal subtype obtained the better response rates to pembrolizumab (47). In our study, basal subtype got higher immune score and higher expression of CD3E, which may enhance the potentiality of CD3E as therapeutic targets. Meanwhile, CXCL12 and CD3E may help patients choose surgery or immunotherapy.

TICs in the TME have been reported to play an essential role in tumor progression and parts of them have been used as available biomarkers. A high neutrophils-lymphocytes ratio (NLR) has been proved associated with adverse outcomes in many solid tumors and has been used as indicator for clinical determinations (48). In this study, we found that the activated and resting dendritic cells were associated with CXCL12, meanwhile, CD4^+^ memory activated and resting T cells and Tregs were negatively correlated with CD3E. Plenty of evidence has shown that TICs, including T cells, natural killer cell, were associated with outcomes of BLCA and may be promising prognostic tools (49). Former IHC researches observed that patients with high levels of CD83^+^ dendritic cells were associated with increased risk of developing MIBC and poor DSS (50, 51). Previous vitro experiment found CD4^+^ T cells were more recruited by BLCA cells than the normal bladder cells, which promoted the BLCA metastasis (52). Both dendritic cells and CD4^+^ T cells were proved associated with BCG induced immune response, which indicated the importance of TICs in BLCA progression and therapy (53). Also, These TICs further proved that CXCL12 and CD3E may be indicators for TME mutation in BLCA.

There are some limitations in present study. For example, our study is based on the TCGA database and larger scale of cases may need to further confirm our study. We roughly divided BLCA patients into luminal and basal subtype but more specific subtypes, such as p53-like, liminal-infiltration, were not lustered. The role of CXCL12 and CD3E in these subtypes needs more exploration. In this study, we used bioinformatics tools to investigate the role of CXCL12 and CD3E in BLCA. However, experiment by vitro and vivo is need to prove the critical effect of CXCL12 and CD3E in TME.

In conclusion, our study shed light on the area of TME in BLCA. We explored the role of TME in BLCA and identified two genes (CXCL12 and CD3E) which may be important divers of immune cells infiltration.

## Data Availability Statement

Publicly available datasets were analyzed in this study. This data can be found here: https://portal.gdc.cancer.gov/.

## Author Contributions

Conceptualization, YL and ZJK; methodology, YL and YCW; validation, PPZ, CX and ZSL; formal analysis, YML; investigation, PPZ; resources, CJH; data curation, YML; writing—original draft preparation, YL; writing—review and editing, YL and YCW; visualization, ZSL; supervision, CJH; project administration, ZJK. All authors contributed to the article and approved the submitted version.

## Funding

The present study was funded by the Key Scientific Item of Henan Province Education Department (21A320037).

## Conflict of Interest

The authors declare that the research was conducted in the absence of any commercial or financial relationships that could be construed as a potential conflict of interest.

## Supplementary Material

Supplementary Figure 1 The result of X-tile software for optimal cut-off value based on immune, stromal, and estimated score. The optimal cut-off value of immune score **(A)**, stromal score **(B)**, and estimated score **(C)**.

Supplementary Figure 2 The result of X-tile software for optimal cut-off value based on the level of level of CXCL12 and CD3E. The optimal cut-off value of the level of CXCL12 **(A)** and CD3E **(B)**.

## Abbreviations

ACKR3, atypical chemokine receptor 3; BCG, Bacillus Calmette–Guerin; BLCA, bladder cancer; CXCR4, CXC chemokine receptor 4; DEGs, differently expressed genes; DSS, disease specific survival; ECM, the extracellular matrix; ERK, extracellular signal-regulated kinase; FC, fold change; FDR, false discovery rate; GO, the gene ontology; GSEA, gene set enrichment analysis; KEGG, the Kyoto Encyclopedia of Genes and Genomes; MIBC, muscle-invasive bladder cancer; NLR, neutrophils-lymphocytes ratio; NMIBC, non-muscle-invasive bladder cancer; OS, overall survival; PD-L1, programmed death ligand 1; PD-1, programmed cell death protein 1; PPI, protein-protein interacting; SDF-1, stromal cell-derived factor-1; TCR, T cell receptor; TICs, tumor-infiltrating cells; TME, tumor microenvironment; Tregs, regulatory T cells; 4F-bTE, 4F-benzoyl-TE14011.
