# Supplementary figures and images for: CXCL12 and CD3E as Indicators for Tumor Microenvironment Modulation in Bladder Cancer and Their Correlations With Immune Infiltration and Molecular Subtypes
Source: Front Oncol. 2021 Mar 4;11:636870. doi: 10.3389/fonc.2021.636870 (PMC7971116; doi:10.3389/fonc.2021.636870)

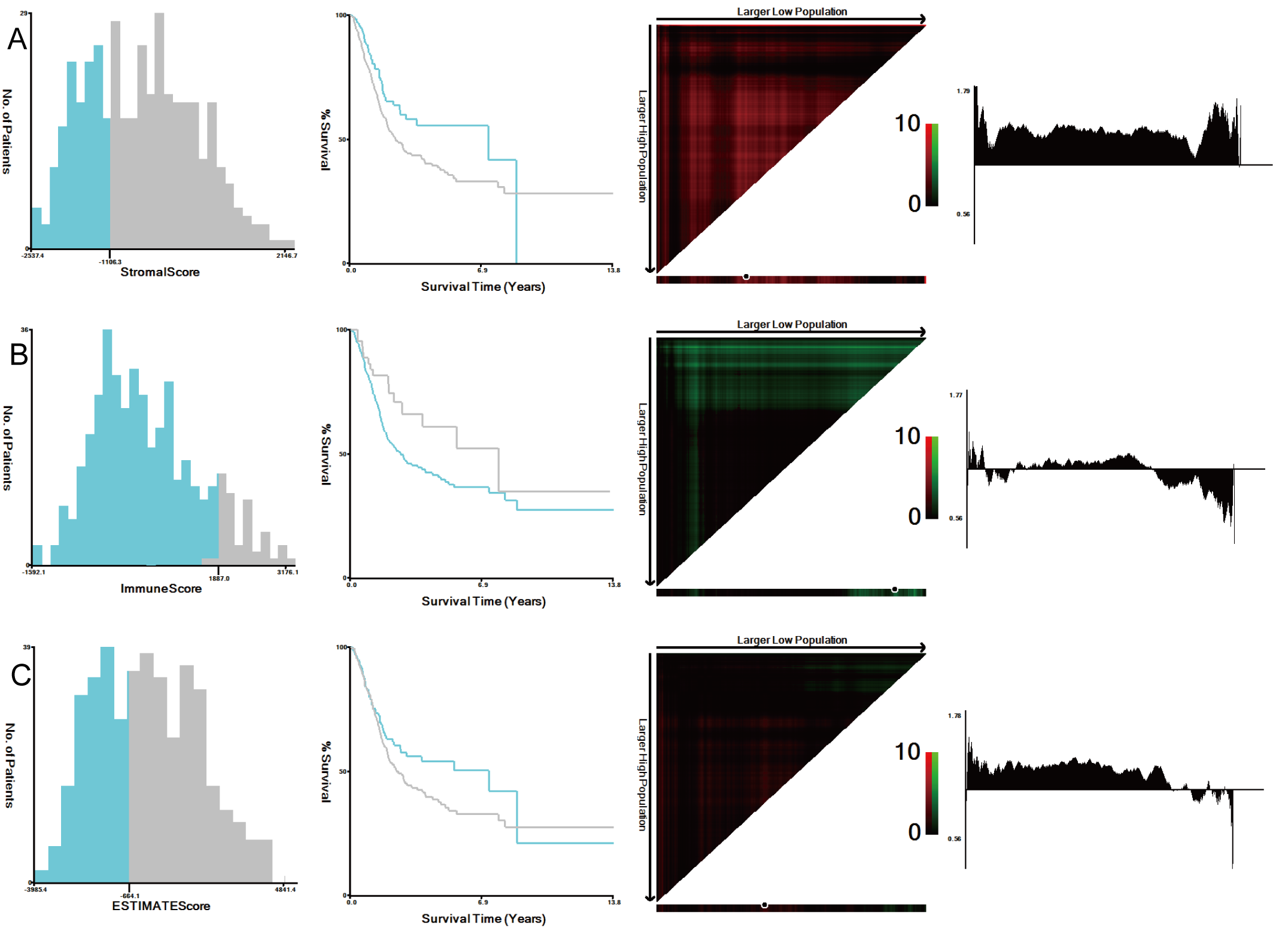

Supplement: Supplementary Figure 1 — The result of X-tile software for optimal cut-off value based on immune, stromal, and estimated score. The optimal cut-off value of immune score (A), stromal score (B), and estimated score (C). [file Image_1.tif]

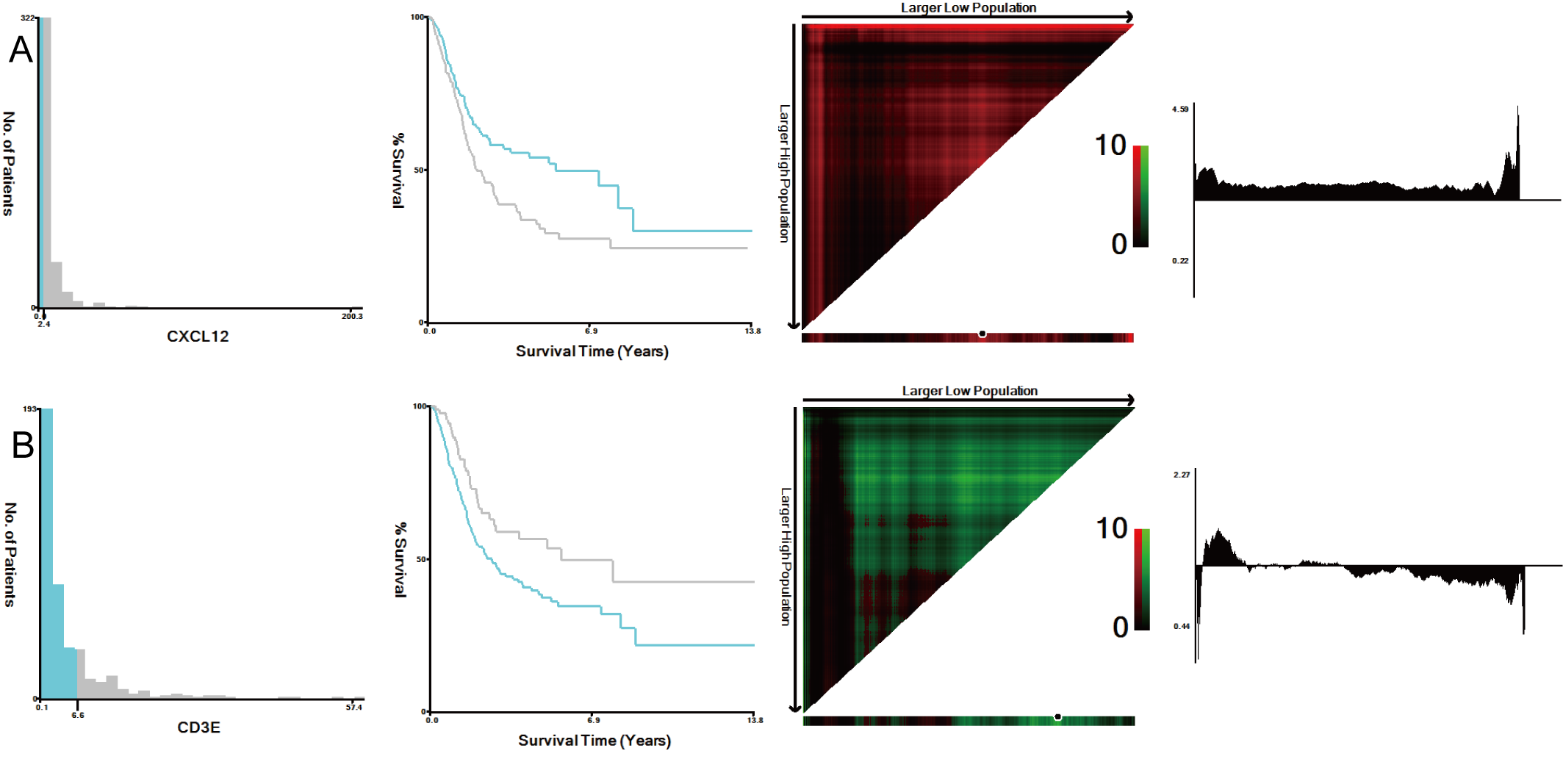

Supplement: Supplementary Figure 2 — The result of X-tile software for optimal cut-off value based on the level of level of CXCL12 and CD3E. The optimal cut-off value of the level of CXCL12 (A) and CD3E (B). [file Image_2.tif]
